# GBR assisted *in situ* Onlay bone grafting for the posterior mandible horizontal ridge augmentation: a case report and literature review

**DOI:** 10.3389/fbioe.2024.1535207

**Published:** 2025-01-10

**Authors:** Mucong Li, Xiuyu Liu, Jing Zhou, Jiaqian You, Sheng Chen, Jian Feng, Xuyan Wei, Hanchi Wang, Yanmin Zhou

**Affiliations:** 1 Hospital of Stomatogy, Jilin University, Changchun, China; 2 Hospital of Stomatology, Guanghua School of Stomatology, Sun Yat-sen University and Guangdong Provincial Key Laboratory of Stomatology, Guangzhou, China

**Keywords:** horizontal ridge augmentation, onlay bone grafting, autologous bone graft, implant restoration, piezoelectric surgery

## Abstract

The posterior mandible is the primary area for occlusal function. However, long-term tooth loss in the posterior mandible often leads to rapid absorption of both buccal and lingual trabecular bone plates and subsequent atrophy of the alveolar ridge. This ultimately results in horizontal bone deficiencies that complicate achieving an optimal three-dimensional placement for dental implants. Conventional techniques employed clinically for horizontal bone augmentation have limited efficacy, cause significant surgical trauma, and require extended treatment duration. Consequently, the selection of an effective and minimally invasive bone augmentation technique for restoring bone width is an essential prerequisite for successful implant restoration in the posterior mandible. This clinical case study presented a treatment approach involving guided bone regeneration (GBR) and *in situ* Onlay grafting for bone level augmentation in the blade-shaped alveolar ridge of the posterior mandible, followed by implant restoration. By rotating the *in situ* sourced bone block, the denser bone volume at the base of the blade-shaped alveolar ridge was transferred to the crest of the alveolar ridge, obviating the necessity for a secondary operative site and mitigating complications such as pain, edema, sensory abnormalities, and nerve injury. Incorporation of trabecular bone within the recipient area enhanced fixation while augmenting vascular supply. A significant increase in bone volume by 1,628.21 mm^3^ was achieved within 7 months postoperatively. Overall, this novel approach offers valuable insights into minimally invasive and stable techniques for alveolar bone augmentation.

## 1 Introduction

The optimal three-dimensional positioning of the implant is a critical determinant for the success of implant restoration and influences the biomechanical factors affecting its longevity. Adequate alveolar bone volume holds considerable significance for achieving this ideal three-dimensional position. The patient’s alveolar bone must not only meet the required height and lateral width for implantation but also ensure a minimum of 1.5–2 mm bone plate thickness on both buccal and lingual aspects of the implant (Chappuis et al., 2018; Chiapasco and Casentini, 2000). The study conducted by Gemma Rubio et al. utilized T-Scan measurements, and demonstrated that the posterior teeth contributed to approximately 82.4% of the total biting force, further highlighting their pivotal role as the primary functional area for biting and bearing the majority of this force (Rubio-Ferrer et al., 2024). Therefore, in implant restoration, posterior implants typically have a larger diameter than anterior ones, demanding greater alveolar bone width. However, tooth loss can result in varying degrees of alveolar ridge resorption, and clinically, more than half of patients fail to meet the criteria for direct implantation. Hence, horizontal bone augmentation surgery is required.

Currently, commonly used and relatively stable bone augmentation techniques in clinical practice include GBR, bone splitting technique, and autogenous bone grafting techniques such as Onlay grafting. Among them, GBR is characterized by lower operational difficulty and easier availability of the grafting material. However, due to the limited spatial support capacity of the collagen membrane, GBR is effective for only modest bone augmentation, making it suitable mainly for mild horizontal ridge deficiencies (Wang et al., 2024). Compared to GBR, the indications for the bone splitting technique are more restricted, as a specific amount of cancellous bone between the buccal and lingual bone plates is required to achieve effectiveness (Atef et al., 2019). Currently, autogenous bone grafting techniques, such as Onlay grafting, are continuously considered the benchmark for ridge reconstruction owing to their exceptional biocompatibility, robust bone guiding, and inducing effects, as well as their osteogenic properties (Sánchez-Labrador et al., 2021). Bone blocks, specifically the mandibular symphysis, lateral ramus, and ramus of the mandible, or those obtained from the ilium, are procured for Onlay grafting, a widely employed technique in addressing horizontal defects within the alveolar bone (Fluegge et al., 2021; Sakaguchi et al., 2023; Wang et al., 2022). However, conventional Onlay bone grafting techniques necessitate the establishment of an additional surgical site, thereby posing potential risks. These risks include wound dehiscence, graft necrosis, infection, hemorrhage, nerve impairment, and sensory aberrations. Therefore, optimizing clinical outcomes in Onlay bone grafting hinges on maximizing graft survival, minimizing resorption, and reducing surgical trauma, which requires careful wound closure. The rapid establishment of blood supply, appropriate microstructural features of the bone fragment (including the composition of cortical and cancellous bone), optimal fixation of the bone fragment, and minimal mechanical stress from soft tissue sources are essential requirements (Sanz-Sanchez et al., 2000; Bayram et al., 2024a).

In the study, the coronal ridge necessitated a horizontal bone augmentation to achieve a quadrilateral bone block with a thin superior aspect and thick inferior aspect. Following slight modification, the block was rotated counterclockwise by 180° and anchored in the original surgical site using titanium pins, in conjunction with conventional GBR techniques. This facilitated the transfer of ample bone volume from the basal region of the coronal ridge to its crest area, eliminating the need for an additional surgical site and minimizing surgical trauma. Meanwhile, it also promoted direct contact between the graft material and recipient cancellous bone, enhancing the stability of fixation and accelerating angiogenesis (Rosenthal and Buchman, 2003).

## 2 Case presentation

### 2.1 Initial status

**FIGURE 1:**
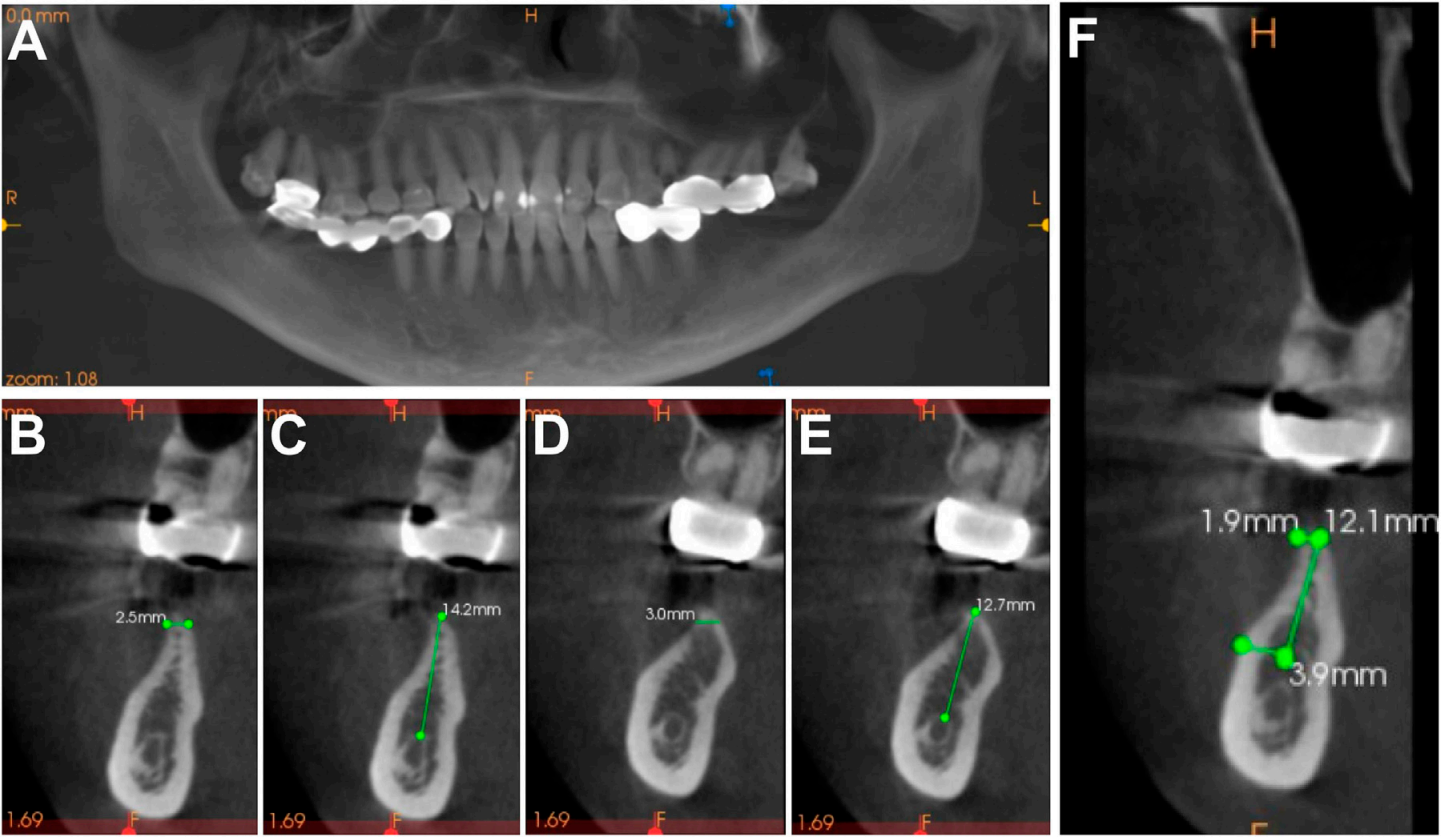
Pre-operative CBCT and surgical plan design **(A)** CBCT panoramic image. **(B)** Available bone height of implant sites #36. **(C)** Alveolar ridge width of implant sites #36 (taking the baseline 1 mm below the dental ridge crest). **(D)** Available bone height of implant sites #37. **(E)** Alveolar ridge width of implant sites #37 (taking the baseline 1 mm below the dental ridge crest). **(F)** *In situ* Onlay bone graft design.

The patient, a 33-year-old female, presented with the chief complaint of “long-term absence of her left lower molar for over 3 years” and expressed a desire for implant restoration. Upon oral examination, missing teeth were observed at sites 36 and 37, accompanied by severe deficiency in alveolar ridge height at the extraction site. The buccal side exhibited approximately 3 mm thickness of keratinized gingiva without any mucosal lesions. Overall, the patient maintained good oral hygiene. Furthermore, cone-beam computed tomography (CBCT, NewTom VGI, QR Srl, Verona, Italy) examination revealed significant horizontal bone resorption with a sharp edge at the site where her left lower jaw was missing teeth. The width of alveolar bone and available bone height at sites 36 and 37 are illustrated in Figures 1A–E.

### 2.2 Treatment plan

The plan, based on the existing bone width, was devised to execute an Onlay grafting procedure with the assistance of GBR. The patient’s preoperative CBCT model was imported into a 3D printer (Objet Connex350, Stratasys, Eden Prairie, Minn) for modeling and scaled printing of the mandible model. Subsequently, a bone harvesting design and simulated surgery were performed. As indicated by CBCT imaging, the vertical distance from the crest of the alveolar ridge to the inferior dental nerve canal in the edentulous area measured approximately 13.45 mm. To minimize neurological complications, a trapezoidal bone block of dimensions 12 mm*12 mm was planned for harvest, featuring a crest thickness of around 1.9 mm and an oblique end thickness of about 3.9 mm (Figure 1F). Prior to surgery, the patient was given thorough instructions on periodontal cleaning to ensure optimal oral hygiene.

### 2.3 Intraoperative phase

**FIGURE 2:**
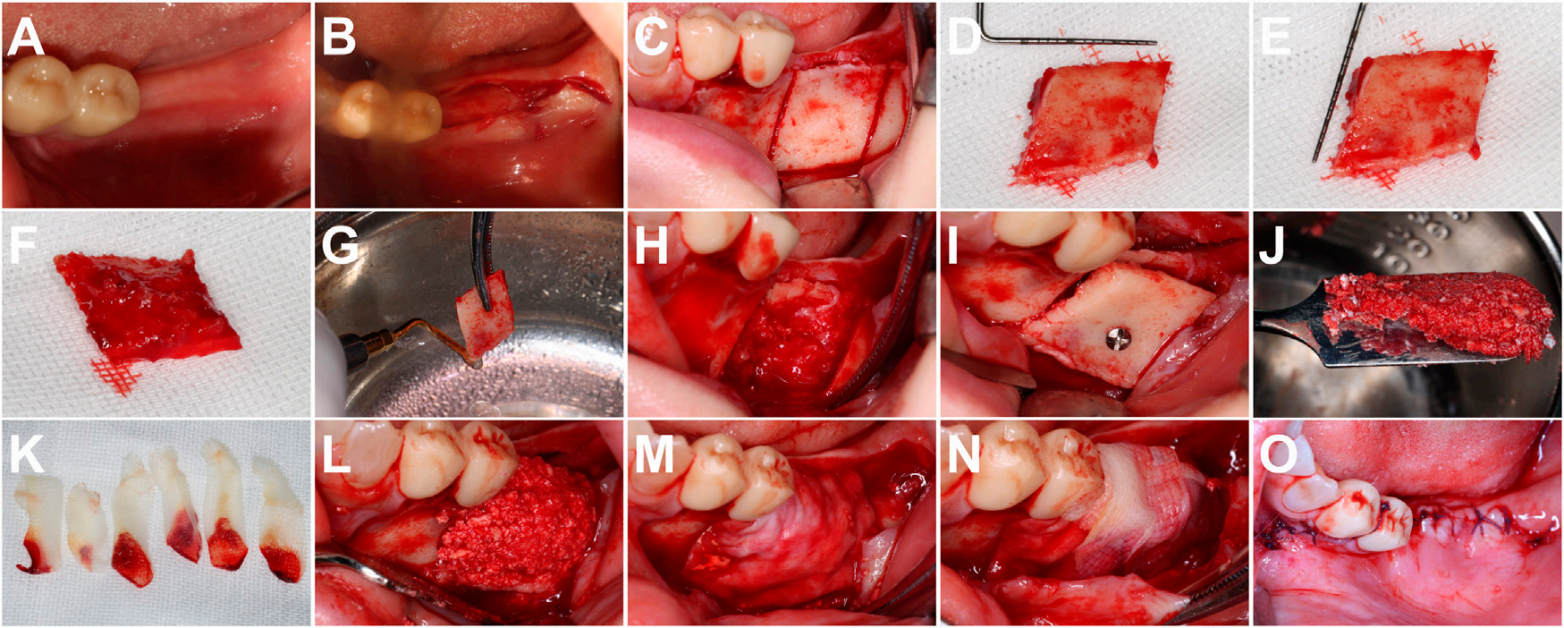
The process of GBR assisted *in situ* Onlay grafting. **(A)** Preoperative morphology of the edentulous ridge. **(B)** Surgical incision. **(C)** Use of mechanical press to cut bone block according to preoperative planning. **(D)** Length measurement of the bone block (approximately 12 mm). **(E)** Width measurement of the bone block (approximately 12 mm). **(F)** Trabecular surface of the grafted bone block. **(G)** Use of mechanical press to trim the edges of the bone block. **(H)** Trabecular structure of the bone after bone extraction. **(I)** Embedding of the grafted bone block into the trabecular structure of the recipient area and fixing it with titanium pins. **(J)** Mixing CGF with Bio-oss bone powder particles. **(K)** Preparing the CGF membrane. **(L)** GBR enhancement: placing Bio-oss bone powder between the block-shaped graft and the jawbone, at discontinuous boundaries and on the surface. **(M)** Covering the collagen membrane. **(N)** Fixing the collagen membrane and covering the CGF membrane sheet. **(O)** Closing the incision with careful suturing.

On the day of the operation, following acquisition of written informed consent, the surgical site was sterilized, and local infiltration anesthesia (1.8 mL of 4% Hydrochloric Aterocaine +1:100,000 Epinephrine) was administered. Subsequently, a 1-cm incision was precisely made on the crest of the alveolar ridge at implant sites #36/37 (FDI World Dental Federation notation), accompanied by an additional incision in the gingival sulcus of the mesial adjacent tooth, along with two vertical incisions. The flaps encompassing both the alveolar ridge crest and buccal mucoperiosteum were meticulously elevated to expose both aspects, i.e., the alveolar ridge and the buccal side of mandibular bone up to external oblique line. Subsequently, utilizing piezoelectric surgical instruments (Guilin Woodpecker Medical Instruments, Guilin, China), a bone block was meticulously separated. The bone block was detached from the base bone using a pericranial bone lifter (JETIP JE4, B&L Biotech, Fairfax, Virginia, United States), and the edges of the bone block were trimmed with a piezoelectric surgical instrument (UL3 tip; Guilin Woodpecker Medical Instrument, Guilin, China). Following that, the bone block was rotated counterclockwise by 180° to ensure that the cancellous surface was oriented towards the defect site upon placement. This rotational placement facilitated the interlocking between the bone graft and cancellous defect interface, ensuring robust stability during transplantation with minimal horizontal slippage. Finally, a 7 mm titanium nail was employed for fixation at its designated transplantation site. No elevation or rotation of the bone block occurred throughout this process of titanium nail implantation. Upon solidification, movement of the fixed bone block was assessed. The gap between the block bone graft and the alveolar bone was filled with a mixture of concentrated growth factor (CGF) and bovine bone mineral graft (Bio-Oss; Geistlich Pharma AG, Wolhusen, Switzerland), while ensuring continuous boundary and surface integration. An absorbable collagen membrane (Bio-Gide; Geistlich Pharma AG, Wolhusen, Switzerland) was subsequently placed to establish an isolation barrier. The absorbable collagen membrane was secured using 3–0 absorbable sutures (PDS, polydioxanone sutures; Ethicon, Somerville, New Jersey, United States), with the dehydrated CGF membrane positioned on top. Finally, buccal flap retraction was performed using a periosteal relaxer, and the wound was closed in a single-stage, tension-free manner utilizing 3–0 absorbable sutures (PDS, polydioxanone sutures; Ethicon, Somerville, New Jersey, United States) (Figure 2). The procedure was complication-free, and the patient did not exhibit any symptoms indicative of inferior alveolar nerve stimulation.

### 2.4 Postsurgical care

0.12% chlorhexidine mouthwash was employed thrice daily for a duration of 2 weeks, and the patient was advised to undergo anti-inflammatory therapy by orally administering amoxicillin at a dosage of 1 g every 12 h for 7 days. Besides, diclofenac sodium (50 mg) should be taken as required for analgesic relief.

**FIGURE 3:**
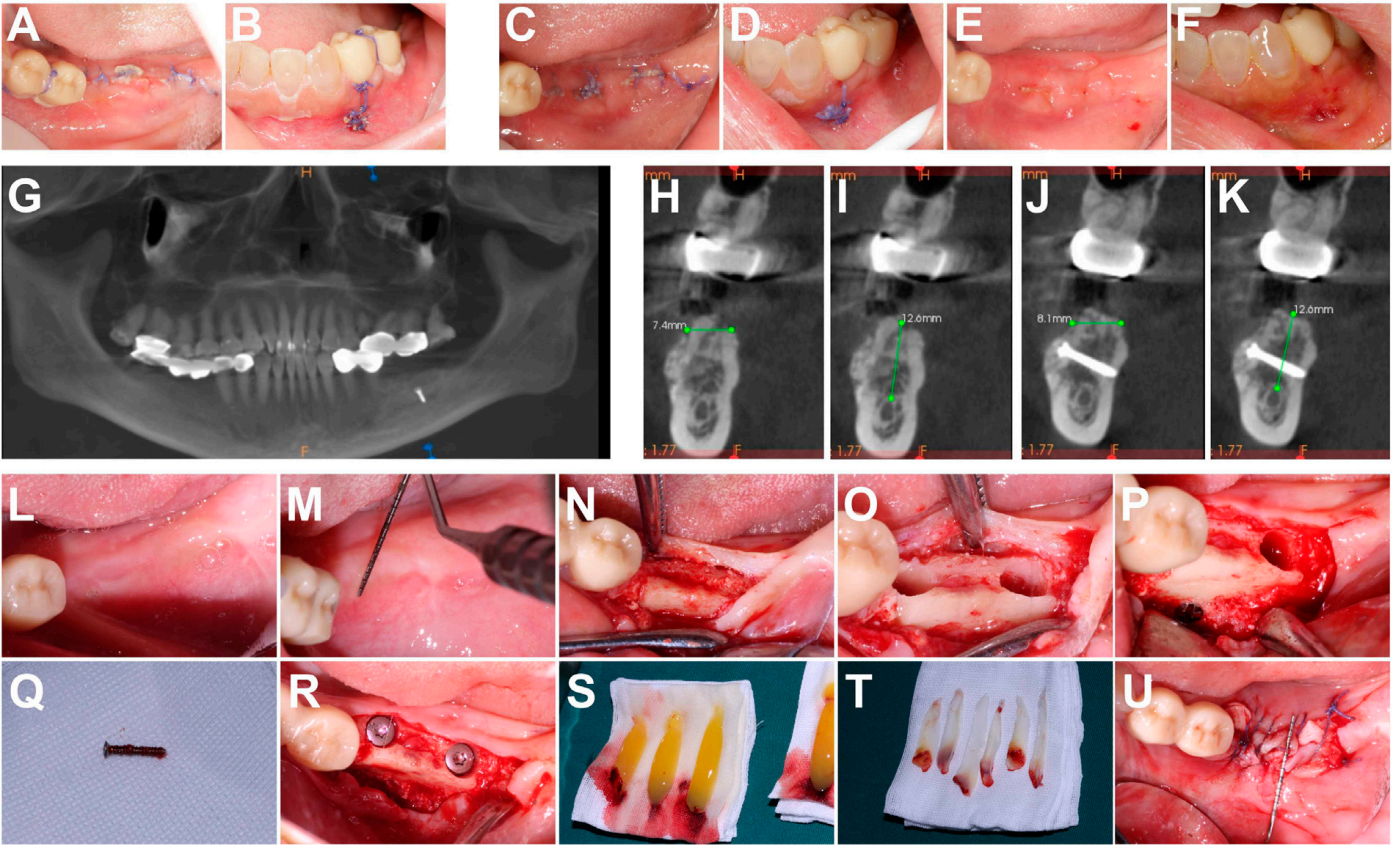
**(A, B)** Postoperative intraoral view on the seventh day. **(C–F)** Postoperative intraoral view on the sixteenth day (after suture removal). **(C, D)** Before suture removal. **(E, F)** After suture removal. **(G–K)** CBCT on the seventh month after the follow-up visit. **(H, I)** Implant sites #36. **(J, K)** Implant sites #37. **(L–U)** Titanium nail removal and implant surgery process.

The patient attended follow-up visits on postoperative days 7 (Figures 3A, B) and 16 (Figures 3C–F), subsequent to suture removal, with no signs of erythema or edema at the surgical site and with favorable mucous membrane healing observed.

### 2.5 Bone augmentation evaluation and implant restoration

At the 7-month follow-up appointment, the patient exhibited well-healed oral mucosa and complete restoration of the alveolar ridge (Figures 3G–K). Subsequently, delayed implant surgery was performed on the same day. Two Straumann Bone Level Tapered Roxolid SLA implants measuring 4.1 mm in diameter and 10 mm in length were placed at implant sites #36/37 upon the removal of the titanium pins. The initial stability of both implants upon placement was measured at 35 Ncm. Furthermore, a CGF membrane was applied over the crest of the alveolar ridge, followed by loose suturing of the wound (Figures 3L–U). Both implants successfully achieved osseointegration during healing.

**FIGURE 4:**
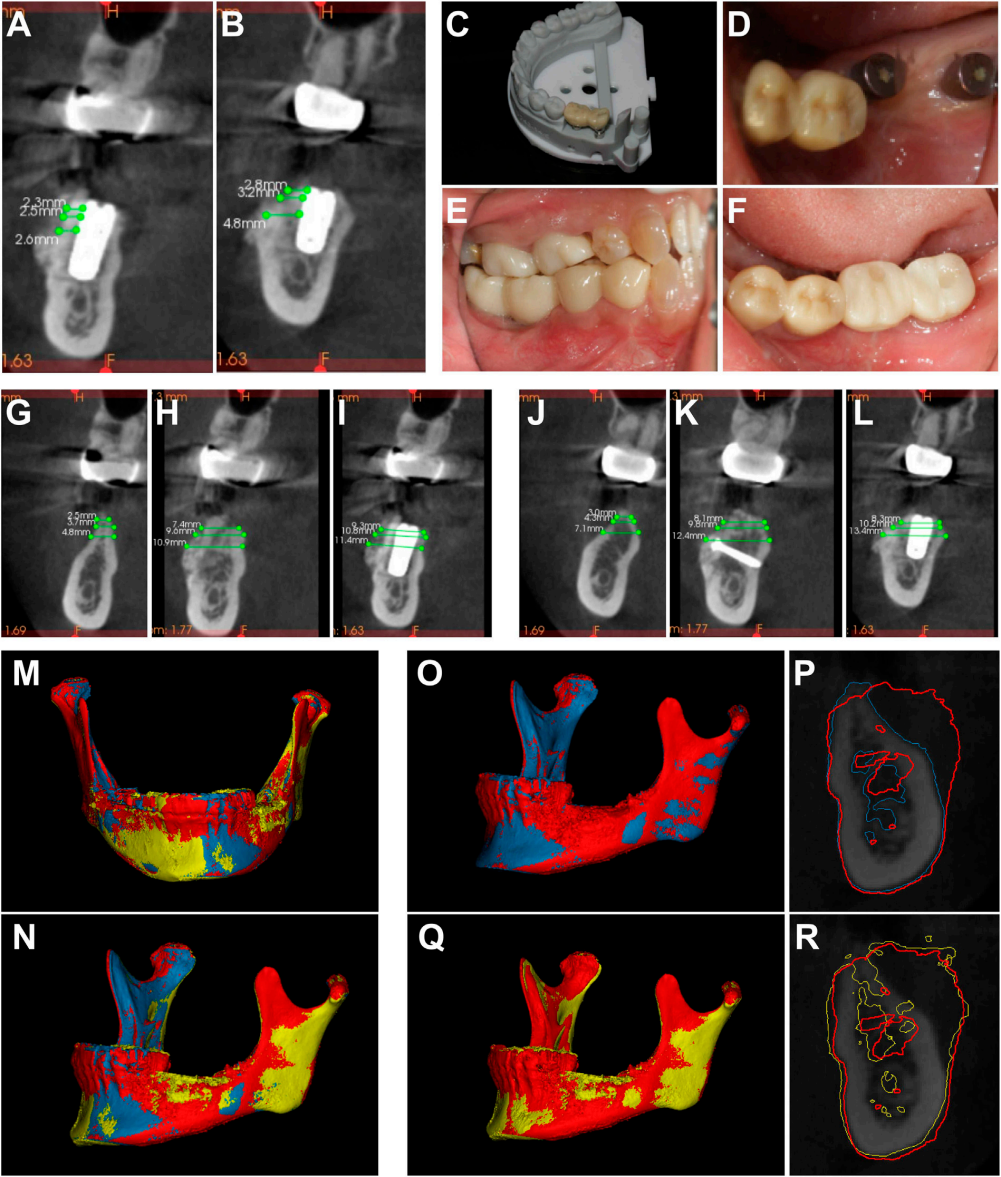
CBCT data of implant sites #36 **(A)** and #37 **(B)** 4 months after implant surgery. **(C–F)** Upper structure completed 4 months after implant surgery. **(G–I)** Alveolar bone width and changes of implant sites #36 **(G)** T0, **(H)** T1, **(I)** T2. **(J–L)** Alveolar bone width and Changes of implant sites #37 **(J)** T0, **(K)** T1, **(L)** T2. **(M, N)** 3D composite images of the mandible based on CBCT data before horizontal bone augmentation (blue), 7 months after horizontal bone augmentation (red), and 4 months after implant placement (yellow). **(O, P)** 3D composite images of implant sites #36 based on CBCT data before horizontal bone augmentation (blue) and 7 months after horizontal bone augmentation (red), along with a comparison of coronal bone volume **(P)**. **(Q, R)** 3D composite images of implant sites #37 based on CBCT data taken at 7 months after horizontal bone augmentation (red) and at 4 months after implant placement (yellow), as well as a comparison of coronal bone volume **(R)**.

At the 4-month follow-up post-implant surgery, a CBCT scan was conducted to check the two implants (Figures 4A, B). Subsequently, a second implant surgery was performed, with two implants (Implant Stability Quotient; ISQ) showing an exceeding value of 75, which met the restoration criteria (Supachaiyakit et al., 2022). Ultimately, digital casting was employed for crown restoration (Figures 4C–F).

The CS 3D Imaging software was utilized to import different CBCTs (CBCT1, CBCT2, and CBCT3), so as to analyze alterations in the width of alveolar bone. Bone thickness (BT) measurements were carried out at 1, 2, and 4 mm levels on the alveolar ridge top for implant sites #36 (Figures 4G–I) and 37 (Figures 4J–L), both before and after horizontal bone augmentation at T0 (prior to horizontal bone augmentation), T1 (7 months post-horizontal bone augmentation), and T2 (4 months after implant placement). The findings are presented in Tables 1, 2.

**TABLE 1:** Alveolar bone width and changes in width of implant sites #36.

BT/Hight	T0	T1	T2	T1-0	T2-1
1 mm	2.5	7.4	9.3	4.9	1.9
2 mm	3.7	9.6	10.8	5.9	1.2
4 mm	4.8	10.9	11.4	5.1	0.5

**TABLE 2:** Alveolar bone width and changes in width of implant sites #37.

BT/Hight	T0	T1	T2	T1-0	T2-1
1 mm	3.0	8.1	8.3	5.1	0.2
2 mm	4.3	9.8	10.2	5.5	0.4
4 mm	7.1	12.4	13.4	5.3	1.0

The CBCT DICOM data of the patient was acquired prior to horizontal bone augmentation, at 7 months post-horizontal bone augmentation, and 4 months upon implant placement using the same equipment. The resulting data was imported into Mimics software (Materialise Mimics V24.0; Materialise; Geomagic 2013.0.1.1206, United States) for generating and fitting 3D models (Figures 4M–R), facilitating the evaluation of the impact of horizontal bone augmentation and subsequent bone resorption following Onlay implantation with GBR support at the site of bone grafting. Compared to pre-implantation bone grafting, the surgical area exhibited an average increase in alveolar bone volume of 1,628.21 mm^3^ over a 7-month follow-up period.

## 3 Discussion

The GBR assisted *in situ* Onlay bone grafting provides a minimally invasive alternative to traditional Onlay bone grafting in the posterior mandible. In recent years, several scholars in clinical practice have utilized autologous bone ring as an alternative to tent nails for horizontal alveolar bone augmentation, achieving promising outcomes. The primary distinction from Onlay bone grafting lies in the use of smaller-volume autologous bone rings as osteogenic and supportive structures, which are inherently constrained by the dimensions of the bone block. Further clinical evidence is required to substantiate their supportive and osteoinductive effects (Chandra et al., 2019; Durrani et al., 2023). In contrast to traditional autogenous block bone grafting for horizontal bone augmentation, which often utilizes the mandible or ilium as donor sites for autogenous bone, this method presents a unique advantage (Elsayed et al., 2024; Manfredini et al., 2022; Mittal et al., 2023). While studies have demonstrated the significant osteogenic potential of this approach, it is frequently accompanied by complications at the donor site, including pain, hemorrhage, infection, nerve injury, and sensory aberrations (Pang et al., 2024; Zhang et al., 2024; Bayram et al., 2024b). In recent years, a relatively abundant bone volume in the basal bone area of the maxillary anterior teeth region has been observed during autogenous bone grafting procedures for vertical bone augmentation (Wang et al., 2022; Yuan et al., 2020; Yang et al., 2021; Wang et al., 2016; Korsch et al., 2022). Horizontal bone augmentation in the anterior region can be accomplished by mobilizing the bone ring or block from the nasal ridge of the maxillary anterior teeth or the mandibular medial suture to the buccal aspect of the alveolar ridge. Specifically, to achieve horizontal bone augmentation on the buccal side of an adjacent edentulous alveolar ridge, Wang et al. implemented a multidisciplinary approach by horizontally transplanting a bone block extracted from an affected tooth’s apical region during endodontic surgery (Wang et al., 2021). While these methods aim to minimize the number of incisions, there still exists a disparity between the bone harvesting area and the implantation site, posing potential risks of injuries and complications associated with the former. To this end, strictly speaking, this technique, which involves transferring relatively abundant bone mass from the basebone area to the crest of the alveolar ridge through bone block displacement, reduces the number of incisions required, effectively condensing the procedure into fewer surgical sites. Furthermore, piezoelectric surgical instruments were hereby employed for bone block harvest and separation, which not only safeguarded soft tissue and nerves but also effectively controlled bleeding while providing an enhanced operative field (Maihemaiti et al., 2023; Rolek, 2024). Furthermore, it could harness the potential of fluid vibration waves to effectively mitigate bacterial levels and yield a desired disinfection outcome (Pavlikova et al., 2011). Additionally, minimizing bone damage during the process of bone collection proves to be more efficacious compared to the application of a ring drill or a bur (Robiony et al., 2007).

The innovative aspect of the *in situ* Onlay bone grafting technique used in conjunction with GBR lies in its efficacy in establishing a spongy-bone interface within the grafting area. Research has demonstrated that this composite structure with cancellous bone on both sides provides enhanced stability for the bone block compared to conventional cortical-cortical or cortical-cancellous interfaces in Onlay grafting procedures. This enhanced stability effectively counteracts rotational forces and micro-motion of the implant, aligning with the principle of primary implant stability as a critical factor for successful bone augmentation (Wang and Boyapati, 2006). In terms of expedited blood vessel regeneration, the trabecular-trabecular interface offers an additional advantage. According to Oppenheimer et al., the local microenvironment of the transplanted bone and the extent of blood vessel regeneration are considered pivotal predictors for graft survival. Among various factors influencing non-vascularized autogenous bone graft outcomes (Oppenheimer et al., 2012), *in situ* bone grafting techniques involve the transfer of bone from one site to another, ensuring the comparable vascular distribution, recipient distribution, as well as immunogenic and genetic characteristics of the donor and recipient areas (Pape et al., 2010). Additionally, a multitude of studies have demonstrated an accelerated and more substantial restoration of blood supply of bone blocks containing trabecular bone compared to those composed solely of cortical bone (Gens et al., 2023; Wang et al., 2023; Lee et al., 2022). Furthermore, the abundance of osteoblasts within the bone marrow cavity, exposure to osteoinductive proteins, and the proliferation and differentiation of mesenchymal stem cells triggered by acute bone injury all contribute significantly to effective vascularization and osseointegration. In this particular case study, a blurred demarcation was observed between the transplanted bone block and alveolar bone upon 7 months of bone augmentation. The transplanted bone block exhibited a healthy coloration, while both implants achieved torque values of 35 N ·cm. Collectively, these results indicate that the fresh wound surface at the harvest site is an ideal receptor area for non-vascularized bone block transplants, offering sufficient blood supply for their viability. This approach performs better in promoting early and rapid osseointegration compared to traditional Onlay bone grafting techniques involving nutrient hole preparation.

However, the *in situ* Onlay bone grafting technique also has certain limitations, as the available bone volume at the *in situ* donor site is constrained compared to that of traditional Onlay bone grafting donor sites (Pape et al., 2010). Furthermore, achieving a precise fit between the transplanted bone block and the margins of the recipient area remains challenging. Thus, additional measures should still be necessarily explored to address this limitation. According to Thomas et al.'s study, combining autologous block bone transplantation with absorbable collagen membrane and demineralized bovine bone granules could yield highly effective and predictable levels of alveolar bone augmentation. The patients experienced an average horizontal bone thickness increase of 4.6 mm, with minimal bone resorption of 0.36 mm observed from the completion of bone augmentation to implant placement (von Arx and Buser, 2006). Similar conclusions were also drawn by Maiorana et al. They revealed that the bone resorption rate in the grafted area treated with ABBM particles was merely 9.3%, while the uncovered area exhibited a higher bone resorption rate of 18.3% (Maiorana et al., 2005). Therefore, Bio-oss demineralized bovine bone granules and collagen membrane were hereby employed to facilitate *in situ* Onlay bone grafting, and thus to augment the grafting efficacy. The primary role of Bio-oss bone powder granules encompasses two aspects. Firstly, they fill the defect area where adequate vascularization enables their involvement in osseointegration and contour remodeling. Secondly, once enveloped by fibers following the absorption of the collagen membrane, the Bio-oss bone powder granules covering the transplanted bone surface act as a barrier against bone resorption. This is because they cannot engage in osseointegration due to an insufficient blood supply (Zerbo et al., 2003). The application of an absorbable collagen membrane in the early phase of bone graft integration can exert a certain influence on maintaining contour and preventing the resorption of block bone graft (Chappuis et al., 2017; Cordaro et al., 2011). Additionally, in cases of limited bone volume, digital tools can enhance surgical precision and personalization. For instance, a precise bone block tailored to the size and contour of the surgical site can be obtained by utilizing preoperative CBCT data to design bone volume parameters and employing 3D printing technology to create a drilling guide. This approach effectively minimizes unnecessary surgical trauma while reducing bone loss associated with reshaping the bone block (Wang et al., 2022).

## 4 Conclusion

The present case report describes the utilization of GBR assisted *in situ* Onlay bone grafting technique for horizontal bone augmentation in the posterior region of the mandible, providing a viable and reliable alternative. By employing autogenous bone blocks *in situ* through trabecular-trabecular contact, this technique enhances the stability and accelerates the blood supply reestablishment, yielding satisfactory bone augmentation effects with minimal surgical trauma. Notably, it exhibits remarkable resistance to resorption during both the healing phase of the bone graft and the post-implant restoration period. Moreover, when combined with GBR technique, the horizontal bone augmentation effect and contour maintenance ability are further augmented. Compared to pre-implantation bone grafting, the surgical area exhibited an average increase in alveolar bone volume of 1,628.21 mm^3^ over a 7-month follow-up period in this case. However, additional clinical cases are still required to substantiate the reliability of this innovative technique.

## Data Availability

The original contributions presented in the study are included in the article/supplementary material, further inquiries can be directed to the corresponding author.
