# Correction: GBR assisted *in situ* Onlay bone grafting for the posterior mandible horizontal ridge augmentation: a case report and literature review

**DOI:** 10.3389/fbioe.2026.1784687

**Published:** 2026-01-21

**Authors:** Mucong Li, Xiuyu Liu, Jing Zhou, Jiaqian You, Sheng Chen, Jian Feng, Xuyan Wei, Hanchi Wang, Yanmin Zhou

**Affiliations:** 1 Hospital of Stomatogy, Jilin University, Changchun, China; 2 Hospital of Stomatology, Guanghua School of Stomatology, Sun Yat-sen University and Guangdong Provincial Key Laboratory of Stomatology, Guangzhou, China

**Keywords:** horizontal ridge augmentation, Onlay bone grafting, autologous bone graft, implant restoration, piezoelectric surgery

The title of this article was erroneously given as: “GBR assisted *in situ* Onlay bone grafting for the posterior maxillary horizontal ridge augmentation: a case report and literature review”. The correct title of the article is “GBR assisted *in situ* Onlay bone grafting for the posterior mandible horizontal ridge augmentation: a case report and literature review”.

The original article has been updated.

